# [Retracted] Knockdown of STMN1 enhances osteosarcoma cell chemosensitivity through inhibition of autophagy

**DOI:** 10.3892/ol.2024.14466

**Published:** 2024-05-20

**Authors:** Zili Wang, Rongzhen He, Hansong Xia, Yu Wei, Song Wu

Oncol Lett 13: 3465–3470, 2017; DOI: 10.3892/ol.2017.5941

Following the publication of this article, a concerned reader has drawn to the Editor's attention that the western blotting data featured in Figs. 2D, 3B, 5A and 5C contained some strikingly similar protein bands, such that these data apparently originated from the same original source where the results from differently performed experiments were intended to have been portrayed; moreover, the GAPDH control western blotting data shown in Fig. 1B were strikingly similar to data that had already appeared in a number of previous publications written by different authors at different research institutes.

Owing to the identification of the apparently overlapping protein bands in Figs. 2, 3 and 5, and given the fact that the contentious data in Fig. 1B of the above article had already been published prior to its submission to *Oncology Letters*, the Editor has decided that this paper should be retracted from the Journal. The authors were asked for an explanation to account for these concerns, but the Editorial Office did not receive a reply. The Editor apologizes to the readership for any inconvenience caused.

